# Risk factors of lower limb cellulitis in a level-two healthcare facility in Cameroon: a case-control study

**DOI:** 10.1186/s12879-017-2519-1

**Published:** 2017-06-12

**Authors:** Tsi Njim, Leopold Ndemnge Aminde, Valirie Ndip Agbor, Louise Daniele Toukam, Sara Saheb Kashaf, Eric O. Ohuma

**Affiliations:** 1 Centre for Tropical Medicine and Global Health, Nuffield Department of Medicine, Old Road Campus, University of Oxford, Oxford, OX3 7BN UK; 2 Health and Human Development Research Group, Douala, Cameroon; 3 School of Public Health, Faculty of Medicine, University of Queensland, Brisbane, Australia; 4 Clinical Research Education, Networking and Consultancy (CRENC), Douala, Littoral Cameroon; 5 Ibal sub – Divisional Hospital, Oku, Northwest Region, Oku, Cameroon; 6 Faculty of Health Sciences, University of Bamenda, Bamenda, Northwest Region, Cameroon; 7 Centre for Statistics in Medicine, Botnar Research Centre, Nuffield Department of Orthopaedics, Rheumatology and Musculoskeletal Sciences University of Oxford, Windmill Road, Oxford, OX3 7LD UK

**Keywords:** Lower limb cellulitis, Risk factors, Disease burden, Cameroon

## Abstract

**Background:**

Cellulitis is a common infection of the skin and subcutaneous tissues. It is associated with significant morbidity from necrosectomies and amputations especially in sub-Saharan Africa. We aimed at identifying the risk factors and burden of lower limb cellulitis to inform preventive strategies in Cameroon.

**Methods:**

This was a hospital–based case-control study carried out in the Bamenda Regional Hospital (BRH) between September 2015 and August 2016. Cases were defined as consenting adults admitted to the surgical unit who presented with a localised area of lower limb erythema, warmth, oedema and pain, associated with fever (temperature ≥ 38 °C) and/or chills of sudden onset. Controls were adults hospitalised for diseases other than cellulitis, necrotising fasciitis, myositis, abscess or other variants of dermo-hypodermitis. Cases and controls were matched (1:2) for age and sex.

**Results:**

Of the 183 participants (61 cases of cellulitis and 122 controls) included in the study, the median age was 52 years [Interquartile range (IQR): 32.5–74.5]. After controlling for potential confounders, obesity [adjusted odds ratio (AOR) = 4.7, 95% CI (1.5–14.7); *p* = 0.009], history of skin disruption [AOR = 12.4 (3.9–39.1); *p* < 0.001], and presence of toe-web intertrigo [AOR = 51.4 (11.7–225.6); *p* < 0.001] were significantly associated with cellulitis. Median hospital stay was longer (14 days [IQR: 6–28]) in cases compared to the controls (3 days [IQR: 2–7]). Among the cases, Streptococci species were the most frequent (*n* = 50, 82%) isolated germ followed by staphylococci species (*n* = 9, 15%). Patients with cellulitis were more likely to undergo necrosectomy (OR: 21.2; 95% CI: 7.6–59.2). Toe-web intertrigo had the highest (48.9%) population attributable risk for cellulitis, followed by history of disruption of skin barrier (37.8%) and obesity (20.6%).

**Conclusion:**

This study showed a high disease burden among patients with cellulitis. While risk factors identified are similar to prior literature, this study provides a contextual evidence-base for clinicians in this region to be more aggressive in management of these risk factors to prevent disease progression and development of cellulitis.

## Background

Cellulitis is a common infection of the skin and subcutaneous tissues that can lead to life-threatening complications [1, 2]. It is an acute, pyogenic infection caused mainly by *Streptococcus pyogenes* or *Staphylococcus aureus*; characterised by an area of cutaneous erythema, warmth and oedema [3, 4]. About 88% of cellulitis occurs in the lower limb [5]. It has a high burden of disease especially in sub-Saharan Africa. According to the Global Burden of Disease 2015 study, cellulitis accounted for 1447 disability adjusted life years (DALYs) in Cameroon and affected mostly men [6]. In South Africa, the annual mortality rate and annual years of healthy life lost per 10,000 people increased by 44.3% and 6.3% respectively between 1990 and 2013. These rates are higher in Zimbabwe with an increase of 75% in annual mortality and 41% increase in years of healthy life lost since 1990 [7]. Previous research elsewhere has shown that cellulitis is associated with a prolonged hospital stay (averagely above four days), increased risk of septic shock and death if nothing is done. Treatment may involve intravenous antibiotics, debridement and amputation in extreme cases [8].

The risk factors of cellulitis can be classified into general and local factors. General risk factors include obesity, diabetes mellitus, history of cellulitis, immunosuppression, lymphedema and peripheral vascular disease while local risk factors include neglected wounds, toe-web intertrigo and leg ulcers [3, 8–10]. A recent multicentre study carried out in eight sub-Saharan African countries including Cameroon identified obesity, lymphedema, voluntary cosmetic depigmentation, neglected traumatic wound and toe-web intertrigo as major risk factors of cellulitis in sub-Saharan Africa [9]. Studies previously carried out in Cameroon have not identified the burden of disease that could be alleviated by reducing the risk factors of cellulitis. The common germs associated with the disease in this setting have not been identified. We carried out this study in a second level healthcare facility in Cameroon to inform clinicians on the risk factors of cellulitis in this area and the common germs involved to guide management. This would also provide policy-makers with data on the associated burden to inform prevention strategies of modifiable risk factors.

## Methods

### Study design and setting

This was a hospital–based case-control study carried out in the Bamenda Regional Hospital (BRH) between the months of September 2015 and August 2016. Bamenda is the capital of the Northwest region of Cameroon with an estimated population of 500,000 inhabitants. The BRH has a total bed capacity of about 600 beds and serves as the main referral hospital in the region, as well as a teaching hospital for the Faculty of Health Sciences, University of Bamenda. The study was conducted mainly at the surgical department of the hospital. The hospital does not have a microbiologic service and superficial swabs with gram staining are the main method of screening for germs from skin specimens.

### Study participants and sampling

All consenting patients hospitalised for cellulitis of the lower limbs during the study period were recruited. For each case, two controls were selected and matched for age and sex. Those who presented with erysipelas (with a localised and well demarcated area of erythema, oedema, redness and pain), necrotising fasciitis of the lower limb, myositis, abscess, other variants of dermo-hypodermitis, or refused to sign an informed consent were excluded from the study.

### Sample size calculation

The sample size was calculated using the formula for difference in proportions [12]:

$$ \mathrm{n}=\left(\mathrm{r}+1/\mathrm{r}\right)\ \left[\left(\mathrm{p}\right)\left(1\hbox{-} \mathrm{p}\right)\ {\left({\mathrm{Z}}_{\mathrm{B}}+{\mathrm{Z}}_{\upalpha 2}\right)}^2\right]/{\left({\mathrm{p}}_1\hbox{-} {\mathrm{p}}_2\right)}^2 $$

Where n is the sample size in the case group, r is the ratio of control to cases (*r* = 2), p is the average proportion exposed. Assuming that the desired power = 80%, Z_B_ = 0.84 and Z_α2_ = 0.96 for a statistical significance at 5%. P_1_ is the proportion of cases exposed and p_2_ is the proportion of controls exposed to one of the risk factors of cellulitis – obesity; which is assumed to be 13% [13]. The minimum odds ratio required to detect an effect will be 3. Therefore, the number of cases required for the study will be 59 and the number of controls – 118.

### Case definition

Cases were defined as patients admitted to the surgical unit who presented with a localised area of lower limb erythema, warmth, oedema and pain, associated with fever (temperature > 38 °C) and/or chills of sudden onset. The cases were hospitalised in the surgical ward after consultation by the surgeon without prior referral as the hospital neither has a dermatology unit nor a dermatologist. Patients who presented with leg ulcers were generally excluded. Only patients who presented with the above case definition and developed an inherent leg ulcer during inpatient monitoring were included in the study.

Our controls were subjects hospitalised for diseases other than necrotising fasciitis, myositis, abscess or other variants of dermo-hypodermitis. Controls were recruited mainly from the same surgical ward where the cases were found except in a few cases where they were obtained from the paediatrics, obstetrics and gynaecology wards.

### Data collection

Data was collected using a pre-structured questionnaire and included: demographic characteristics (age, sex, marital status, occupational status, level of education), clinical profile (diseased systems for controls), general risk factors for cellulitis [histories of diabetes, hypertension and human immunodeficiency virus (HIV)], local risk factors of cellulitis [past history of surgery to the lower limb and history of trauma or disruption of skin barrier (recent trauma of the lower limb leading to break of skin with or without break in subcutaneous tissues)], social history (histories of alcohol consumption, tobacco smoking and use of other recreational drugs) and length of hospital stay. The participants were then examined clinically for presence of toe-web intertrigo (mild scaling to an exudative, macerated or erosive process of the toe web space) [14], their heights and weights were recorded after measurement. Samples were collected from the toe-web and/or an ulcer of the affected limb of participants with cellulitis and sent to the laboratory for gram staining.

Obesity was defined as a body mass index (BMI) of 30 kg/m^2^ and above [15]. We defined alcohol misuse as a consumption of more than 11.2 g/l (14 units) of alcohol per week. History of HIV was sub-categorised into; severe immunodeficiency (CD4+ count <200 cells/mm^3^) or not (CD4+ ≥200 cells/mm^3^) based on the most recent measurement within a 3 month period [16], and the duration after diagnosis at which a person living with HIV is at risk of soft tissue infection (≥ 4 years) or not (< 4 years) [17]. Also, the disease burden was assessed by the length of hospital stay, need for necrosectomy and eventual amputation.

### Ethical considerations

This study was approved by the ethical review board of the Bamenda Regional Hospital and authorisation was granted by the Director of the said institution prior to the study. A signed informed consent was required from participants ≥18 years or from the guardians of participants <18 years after detailed explanation about the study in their language of choice: English, French or Pidgin (the local lingua franca), before they were enrolled into the study. All information collected from our study was treated as confidential.

### Statistical analysis

All data were entered using Epi info version 7.0.8.3 and analysis was done with STATA version 12.1. Categorical variables were presented as frequencies and proportions. Continuous variables were presented as means and medians where applicable. Categorical variables were compared using a Chi square test and Odds ratios (OR) were used to assess the degree of associations. Variables with a *p* value <0.1 on univariate analysis were included in the multivariate logistic regression analysis model in a stepwise fashion and removed if the *p* value was >0.05, to identify risk factors for cellulitis. Statistical significance was considered at the 5% level for the multivariable analysis.

A sensitivity analysis was done excluding paediatric patients (age ≤ 18 years [18]) by repeating the above procedures.

Finally, we calculated the population-attributable risk (PAR) for factors which were associated with cellulitis to estimate the proportion of preventable disease if these risk factors were reduced or eliminated. Based on the assumption that the controls are representative of the general population in which cellulitis is an infrequent outcome, we calculated the PAR using the formula [19]:

$$ PAR=\frac{P\left( RR-1\right)}{P\left( RR-1\right)+1} x100\% $$

P = prevalence of the given risk factor in the control group and RR = Risk ratio.

## Results

A total of 183 participants were included in this study (61 cases of cellulitis and 122 controls). Majority of the cases (*n* = 40) and controls (*n* = 80) were female. The median age of cases and controls was 52.0 years [Interquartile range (IQR) = 32.5–74.5]. About 50% of our study participants, both cases and controls, were unemployed.

The admission diagnosis for control participants was: infectious diseases in 49 (40%), cardiovascular diseases in 19 (16%), neoplasms in 10 (8%) and other diseases in 46 (37.7%) (Table 1).

**Table 1:** Socio-demographic characteristics of cases of cellulitis and controls at the Bamenda Regional Hospital, September 2015 to August 2016

Characteristic	*N* (%) of case (*n* = 61)	*N* (%) of controls (*n* = 122)	*p* value
1. Socio-demographic characteristics			
Age (years)^a^			
15–24	10 (16.4)	20 (16.4)	
25–34	7 (11.5)	14 (11.5)	
35–44	6 (9.8)	12 (9.8)	
45–54	10 (16.4)	20 (16.4)	
55–64	6 (9.8)	12 (9.8)	
65–74	7 (11.5)	14 (11.5)	
75–84	9 (14.8)	18 (14.8)	
≥ 85	6 (9.8)	12 (9.8)	
Marital status			
Married	40 (65.6)	79 (64.8)	0.913
Single	21 (34.4)	43 (35.2)	
Occupation			
Unemployed	33 (54.1)	52 (42.6)	0.142
Employed	28 (45.9)	70 (57.4)	
Gender^a^			
Male	21 (34.4)	42 (34.4)	
Female	40 (65.6)	80 (65.6)	
Level of education			
None	22 (36.1)	33 (27.0)	0.383
Primary	21 (34.4)	44 (36.1)	
Secondary	14 (22.9)	28 (23.0)	
Tertiary	4 (6.6)	17 (13.9)	

^a^ *p* value not provided because cases were matched for Age and Gender

In the univariate analysis; obesity (OR = 3.0; 95% CI = 1.5–6.0; *p* = 0.002), history of diabetes (OR = 2.5; 95% CI = 1.1–5.8; *p* = 0.033), alcohol consumption (OR = 3.0; 95% CI = 1.5–5.9, *p* = 0.001) and alcohol misuse (OR = 11.3; 95% CI = 1.3–98.3; *p* < 0.001), history of disruption of skin barrier (OR = 8.4; 95% CI = 4.0–17.6; *p* < 0.001), history of surgery to the lower limb (OR = 10.4; 95% CI = 2.2–49.7; *p* < 0.001) and presence of toe-web intertrigo (OR = 34.5; 95% CI = 12.3–97.0; *p* < 0.001) were statistically significantly associated with cellulitis (Table 2). In multivariable analysis; obesity (AOR = 4.7, 95% CI (1.5–14.7); *p* = 0.009), history of skin disruption [AOR = 12.4 (3.9–39.1); *p* < 0.001], and presence of toe-web intertrigo (AOR = 51.4 (11.7–225.6); *p* < 0.001) remained significant risk factors for cellulitis (Table 2).

**Table 2:** Risk factors for lower limb cellulitis in the Regional Hospital Bamenda, Cameroon, September 2015 to August 2016

Risk factors	No. (%) cases (*n* = 61)	No. (%) controls (*n* = 122)	OR (95% CI)	*p* value	Adjusted OR (95% CI)	*P* value
*Social factors*						
Marital status						
Married	40 (33.6)	79 (66.4)	1.0 (0.5–2.0)	0.524		
Single	21 (32.8)	43 (67.2)				
Occupation						
Unemployed	33 (38.8)	52 (61.2)	0.6 (0.3–1.2)	0.142		
Employed	28 (28.6)	70 (71.4)				
General factors						
Obesity						
Yes	27 (56.3)	21 (43.7)	3.0 (1.5–6.0)	0.002	4.7 (1.5–14.7)	0.009
No	34 (30.1)	79 (69.9)				
History of diabetes						
Yes	13 (52.0)	12 (48.0)	2.5 (1.1–5.8)	0.033	0.9 (0.2–3.8)	0.846
No	48 (30.4)	110 (69.6)				
History of cellulitis						
Yes	3 (50.0)	3 (50.0)	2.1 (0.4–10.5)	0.379		
No	58 (32.8)	119 (67.2)				
History of hypertension						
Yes	21 (44.7)	26 (55.3)	1.9 (1.0–3.8)	0.056	1.3 (0.4–3.7)	0.687
No	40 (29.4)	96 (70.6)				
History of HIV						
Yes	6 (21.4)	22 (78.6)	0.5 (0.2–1.4)	0.170		
No	53 (34.6)	100 (65.4)				
Duration of HIV (years)						
≥ 4	4 (23.5)	13 (76.5)	1.1 (0.2–7.4)	0.940		
< 4	2 (22.2)	7 (77.8)				
CD4+ count (cells/mm^3^)						
< 200	1 (8.3)	11 (91.7)	0.2 (0.0–1.7)	0.099		
≥ 200	5 (35.7)	9 (64.3)				
Alcohol consumption						
Yes	25 (52.1)	23 (47.9)	3.0 (1.5–5.9)	0.001	2.4 (0.7–7.9)	0.152
No	36 (26.7)	99 (73.3)				
Alcohol misuse^b^						
Yes	16 (94.1)	1 (5.9)	11.3 (1.3–98.3)	<0.001	-	-
No	9 (31.0)	20 (69.0)				
History of smoking						
Yes	9 (47.4)	10 (52.6)	1.9 (0.7–5.1)	0.170		
No	52 (31.7)	112 (68.3)				
Current smoker						
Yes	3 (30.0)	7 (70.0)	0.9 (0.2–3.5)	0.847		
No	56 (32.9)	114 (67.1)				
*Local factors*						
History of disruption of skin barrier^*a*^						
Yes	33 (68.8)	15 (31.2)	8.4 (4.0–17.6)	<0.001	12.4 (3.9–39.1)	<0.001
No	28 (20.7)	107 (79.3)				
History of surgery to the lower limb						
Yes	9 (81.8)	2 (18.2)	10.4 (2.2–49.7)	<0.001	4.6 (0.4–51.8)	0.212
No	52 (30.2)	120 (69.8)				
Presence of toe-web intertrigo						
Yes	37 (88.1)	5 (11.9)	34.5 (12.3–97.0)	<0.001	51.4 (11.7–225.6)	<0.001
No	24 (17.6)	112 (82.4)				

*n* number of cases, *OR* Odds ratio, *CI* Confidence interval

^*a*^At most four weeks before onset of cellulitis

^b^omitted from multivariate regression model due to multicollinearity

A sensitivity analysis done excluding the paediatric patients (*n* = 27) yielded similar effect sizes.

Of the 61 samples collected from the toe-webs and/or ulcers for gram staining, 50 (82%) were positive for streptococci species, 9 (15%) for staphylococci species, while a dual infection with the above bacteria species was noticed in 2 (3%) samples. Cellulitis was associated with a median hospital stay of 14 (IQR: 6–28) days, compared to 3 (IQR = 2–7) days for the controls. Necrosectomy was a significant complication of cellulitis: the odds of undergoing necrosectomy in participants with cellulitis were 21 times higher than their counterparts without cellulitis (*p* < 0.001). The median hospital stay for patients with cellulitis who underwent a necrosectomy was longer (21 days) than for patients who did not have cellulitis (4 days) (Table 3). Presence of toe-web intertrigo was associated with the highest PAR (48.9%), followed by history of disruption of skin barrier (37.8%) then obesity (20.6%).

**Table 3:** Complications of lower limb cellulitis in the Regional Hospital Bamenda, September 2015 to August 2016

Complication	Case (*n* = 61) *n* (%)	OR (95% CI)	*P* –value
Amputation			
Yes	3 (4.92%)	3.1 (0.5–19.1)	0.207
No	58 (95.08%)		
Necrosectomy			
Yes	29 (47.54%)	21.2 (7.6–59.2)	<0.001
No	32 (52.46%)		

*n* number of cases, *OR* Odds ratio, *CI* Confidence interval

## Discussion

This study determined the risk factors and burden of cellulitis in a second level healthcare facility in Cameroon. Obesity, a history of disruption in skin barrier and presence of toe-web intertrigo were shown to be risk factors of cellulitis.

Obesity, diabetes mellitus, history of cellulitis, immunosuppression, lymphedema, peripheral vascular disease, voluntary cosmetic depigmentation, disruption in cutaneous barrier, toe-web intertrigo and leg ulcers have been identified as risk factors of cellulitis in previous studies [3, 8–10]; with toe-web intertrigo and disruption in skin barrier consistently reported as risk factors of cellulitis. In this study, disruption in the skin barrier and presence of toe-web intertrigo were strongly associated with cellulitis reaffirming the role played by local risk factors in the development of cellulitis of the leg. Diabetes as well as infection with HIV cause immune suppression, which then favours development of cellulitis. However, contrary to previous reports which have found consistent relationships between cellulitis and these two conditions [12, 13], we did not find such associations in our study. Other studies by Pitché et al. [9], Dupuy et al. [10] and Björnsdóttir et al. [20] similarly did not find an association between cellulitis and these two conditions.

Every sample collected from the toe-web and/or ulcer of the affected limb of participants with cellulitis was positive for at least one bacteria genus. Ninety-seven percent of the samples collected from the toe-web of participants with cellulitis were mono-microbial with either the presence of streptococci or staphylococci species; a majority being infected with the former. Some studies have demonstrated streptococci - especially group A beta-haemolytic streptococci, as major causative agents of bacterial dermo-hypodermal infections [4, 20]. This finding strengthens the role played by streptococci in the pathogenesis of cellulitis and encourages the use of anti-streptococci antibiotics in the treatment of this pathology. The role of staphylococci should not be overlooked as other studies have consistently identified this pathogen in participants with cellulitis [3, 20]. Nevertheless, our study failed to identify gram-negative bacteria reported by other studies [3].

Cellulitis was associated with a length of hospital stay of 14 days. On average, they stayed 11 days longer in hospital than the controls. An average length of hospital stay of 4 to 11 days has been reported by previous studies [5, 14–16]. In a population where about 50% of the participants are unemployed and are thus dependent on subsistence farming to meet their daily needs, these long hospital stays lead to lost-productivity and income; especially for Cameroon with absence of universal health insurance schemes and cost of illness borne entirely by patients. This further worsens a vicious cycle of poverty and disease especially for the vulnerable populations.

Participants with cellulitis had significantly higher odds of undergoing a necrosectomy compared with some of the controls who presented with deep wounds following road traffic accidents and infected burns. It is possible that indiscriminate prescription of antibiotics as well as high use for self-medication at home with the final endpoint, amongst others, of antibiotic resistance; likely contributed to observed poor response to treatment, need for necrosectomy and thus long hospital stays.

Finally, for public health intervention measures, we calculated the PAR of each independent risk factor. Interestingly, these risk factors (presence of toe-web intertrigo, history of disruption in skin barrier and obesity) are modifiable. Presence of toe-web intertrigo had the highest PAR; highlighting the role of toe-web intertrigo in the pathogenesis of cellulitis. Aggressive management of this condition alongside other causes of skin infections and leg ulcers by clinicians is required to curb the burden associated with these conditions. Unfortunately, only a minority of patients with toe-web intertrigo are receiving treatment [11, 21]. Also, history of disruption in skin barrier was associated with the second highest PAR; stressing on the importance of this risk factor in the development of cellulitis.

### Limitations and strengths of the study

The predictors in this study were based on the potential of the participants to recall their exposures to the risk factors (history of disruption of skin barrier, smoking and alcohol intake), thus subjecting the study to recall bias. The use of cultures is more appropriate to determine speciation of bacteria species. Due to the lack of appropriate platforms, we used superficial swabs and Gram staining for this study which might be less reliable. The overall accuracy of superficial swabs compared with deep tissue specimens is 73% [22]. Being a single-centre and hospital-based study; our findings may not be generalisable to the rest of the country. The wide confidence intervals obtained could be explained by the relatively small sample size indicating the need for much larger studies across the country.

However, this is the lone study in the Northwest region of the country which has explored the risk factors of cellulitis and its associated burden and provided local and context-specific evidence for clinical and public health practice.

## Conclusion

This study showed a high disease burden associated with cellulitis. Thorough physical examination by clinicians in this region to identify potential risk factors observed in this study, amongst others, is paramount to prevent disease progression; and is also necessary for prompt and accurate diagnosis, investigation and management of cellulitis. For a resource-constrained setting with limited access and/or affordability of culture like ours, empiric antibiotics targeting commonest local germs remain a best alternative. Finally, we recommend larger multicentre studies to better characterise the burden of cellulitis in the country.

## Abbreviations

AOR: Adjusted Odds ratio
BMI: Body Mass Index
CI: Confidence interval
OR: Odds ratio
PAR: Population-attributable risk
RR: Relative risk
